# The effects of solar radiation modification on solar and wind resource and power generation in the Caribbean

**DOI:** 10.1371/journal.pone.0325226

**Published:** 2025-06-05

**Authors:** Matthew St. Michael Williams, Leonardo A. Clarke, Randy Koon Koon, Michael A. Taylor, Jayaka D. Campbell, Tannecia S. Stephenson

**Affiliations:** 1 Climate Studies Group Mona (CSGM), Department of Physics, University of the West Indies, Kingston, Jamaica; 2 School of Engineering, College of Engineering, Environment and Science, Coventry University, Coventry, United Kingdom; Yalova University, TÜRKIYE

## Abstract

The slow pace of global mitigation efforts has led to increased interest in Solar Radiation Modification (SRM) as a means for rapidly and artificially cooling the planet. Deploying SRM technologies, however, may directly alter renewable energy resources. This makes it a concern for Caribbean countries which are investing heavily in Variable Renewable Energy (VRE) to reduce their reliance on imported energy and meet climate change mitigation goals. In this study, solar irradiance output is extracted from the HadGEM2-ES global climate model run using the G4 (Stratospheric Aerosol Injection) SRM scenario from the Geoengineering Model Intercomparison Project (GeoMIP). The data is extracted for two future time periods corresponding to when global surface temperatures are projected to be 1.5 ∘C and 2.0 ∘C  above pre-industrial levels using the HadGEM2-ES run under the Representative Concentration Pathway 4.5 (RCP4.5) scenario. Wind speed data are similarly extracted but for the HadGEM2-ES run using the G4, as well as the G4cdnc and G4seasalt (Marine Cloud Brightening) GeoMIP scenarios. The solar and wind data are used to evaluate changes in solar photovoltaic (PV) and wind farm power generation in the Caribbean in future ‘SRM versus non-SRM worlds’. Solar irradiance resources and PV energy generation generally decrease under SRM compared to RCP4.5. The highest modelled mean change in PV generation across the region is, however, generally small, e.g., a maximum change of −1.37% for May-July for years corresponding to a 2.0 ∘C world. In contrast, wind power generation under SRM compared to RCP4.5 generally show large increases which are both seasonally and SRM technology dependent. For a 67m turbine, the highest regional wind generation change was +64.72 % for December-February under G4cdnc in a 2.0 ∘C world but −0.56%  under G4 in a 2.0 ∘C world for the same period. For a 100 m turbine, the highest change was an increase of 89.75% for August-September under G4cdnc in a 1.5 ∘C world and a decrease of −4.11% for December-January under G4 in a 2.0 ∘C world. Marine Cloud Brightening-based SRM scenarios (G4cdnc and G4SeaSalt) produce the most consistent spatial increases in wind power resources and generation compared to Stratospheric Aerosol Injection (G4). The findings of this study corroborate and present new findings about potential SRM induced changes on the VRE resources considered important for the Caribbean’s future development. It is therefore important that the region’s energy sector engage in the global discussions underway on the future use of SRM as a strategy for limiting future global warming.

## Section 1: Introduction

### 1.1 Background

Small Island Developing States (SIDS) are amongst the most vulnerable to the impacts of climate change. Within this group, Caribbean countries have experienced 60% of the climate-related disasters affecting SIDS over the past 50 years [1]. Impacts include coastal erosion, flooding, heat stress, increased pressure on water and food resources, loss of livelihoods, loss of lives, and reduced economic productivity [1–5]. Projections indicate that these challenges will intensify as climate change continues [6–8].

Global strategies to address the climate crisis have primarily focused on adaptation and mitigation through greenhouse gas (GHG) reduction [9,10]. However, in recent years, Solar Radiation Modification (SRM) has been proposed as an additional option for limiting the climate change threat. SRM involves rapidly cooling the planet through artificial means by modifying incoming solar or outgoing terrestrial radiation. With the 1.5 ∘C global warming threshold strongly advocated by SIDS [11] nearing exceedance on the current trajectory of global emissions, SRM is being suggested as a complement to GHG reduction strategies for limiting global temperature rise [12].

Proposed SRM techniques include Stratospheric Aerosol Injection (SAI), which involves dispersing reflective particles into the lower stratosphere [13]; Marine Cloud Brightening (MCB), which increases the reflectivity of low-level ocean clouds by spraying them with seawater droplets [14]; Cirrus Cloud Thinning (CCT) or thinning high-level cloud cover to enhance terrestrial radiation loss [15]; and Space-Based Reflectors (SBR) which reflect a portion of incoming solar radiation [12]. Despite the global implications, SIDS have largely been excluded from SRM discussions until recent years [16].

Most of the SRM research to date is based on computational simulations rather than experimentation due to uncertainties about physical feedback mechanisms, the lack of governance frameworks for large-scale deployment, and ethical considerations [17–19]. These simulation studies are predominantly led by developed countries with advance computational capabilities. Initiatives such as the Geoengineering Model Intercomparison Project (GeoMIP) [20] have established standardized scenarios to facilitate intercomparison of model results. The modelled scenarios range from global reductions in solar radiation, such as the G1 scenario, to threshold-based interventions like G6, which aim to prevent overshooting specific temperature thresholds [21].

SRM studies have examined the global, hemispheric, and regional impact of its deployment [22–28]. Notwithstanding, there is a gap in research focused on developing countries. Only two studies have examined SRM impacts on the Caribbean [29,30]. The Caribbean studies have emerged from the DEGREES initiative which seeks to build the capacity of developing countries to evaluate SRM. The first Caribbean study explored changes in the mean near-surface temperature and rainfall under two GeoMIP phase 1 SAI-based scenarios (G3 and G4) compared to a non-SRM scenario (RCP4.5) [29]. The study revealed that while SRM can delay global temperature thresholds, such as 1.5 ∘C, by 8–33 years compared to RCP4.5, it also induces widespread cooling and drying in the Caribbean. This cooling-drying relationship due to SRM has also been observed in other regions of the world [31,32].

### 1.2 The rationale and research gap

There is an urgent need for more modelling studies to characterize SRM’s impact on the Caribbean. This study addresses this gap by investigating SRM’s potential effects on the region’s future energy security through its impact on Variable Renewable Energy (VRE) resources. It builds on preliminary work by [30], which focused on SRM’s influence on inter-island VRE power complementarity.

Energy security is defined as the “uninterrupted availability of energy sources at an affordable price” [33]. The Caribbean region heavily relies on imported fossil fuels, which accounted for 85% of its energy supply and 8% of its Gross Domestic Product in 2019. This has been identified as a significant barrier to economic growth [34–36]. As a result, most Caribbean nations have adopted dual goals of improving energy resilience and reducing GHG emissions by transitioning to VRE technologies such as solar and wind power [36,37].

SRM-induced changes in climate will alter the resources upon which VRE technologies depend. This will have economic implications for Caribbean countries which already lack sufficient international climate financing [38]. Understanding the potential impact of SRM is necessary to facilitate expanded adoption of solar and wind to meet regional energy goals while accounting for uncertainties that may result from its deployment. While existing literature extensively examines the impacts of climate change on VREs [39–44], there is need to expand research to include SRM impacts.

To date, only two studies have explored the intersection of SRM and VRE. Both focus on SRM’s global effects on solar power. [45] projected a 5.9% global reduction in concentrated solar power (CSP) under fixed Stratospheric Sulphate Injection (SSI) of 10Tg of SO_2_ per year (comparable to GeoMIP G4) for 2040–2059. This reduction reaches up to 10−20 % in equatorial and low-latitude regions, with a 0.89 % global reduction for Solar PV. A second study [46] examined a gradually increasing SSI scenario (G6sulfur) and predicts CSP reduction of 7.6 %−7.8 % and solar PV reductions of 4.2−6.9 % for 2090–2099. Notably, neither study explored SRM’s impact on wind resources or examined regional implications.

### 1.3 Aim of the study

This study seeks to advance the literature by:

1. Modelling and assessing the potential impacts of SRM on wind resources and power, focusing on the Caribbean.
2. Investigating the potential impact of MCB-based SRM on VRE resources using GeoMIP outputs and open-source tools.
3. Evaluating the impact of SRM on solar resources and solar power potential, in the Caribbean.

This is the first study examining the potential impact of SRM on an important sector for the Caribbean. Using outputs from a climate model run using three GeoMIP SRM scenarios and one non-SRM scenario, the study employs Python-based models to simulate solar and wind energy potential. Results are analyzed for two future periods corresponding to global temperature increases of 1.5 ∘C, and 2.0 ∘C above pre-industrial levels in a non-SRM climate change world (RCP4.5). The methodology, study domain, and datasets are detailed in Section 2, with results and findings presented in Sections 3 and 4.

## Section 2: Study domain, datasets and methodology

### 2.1 Study domain

**Fig 1:**
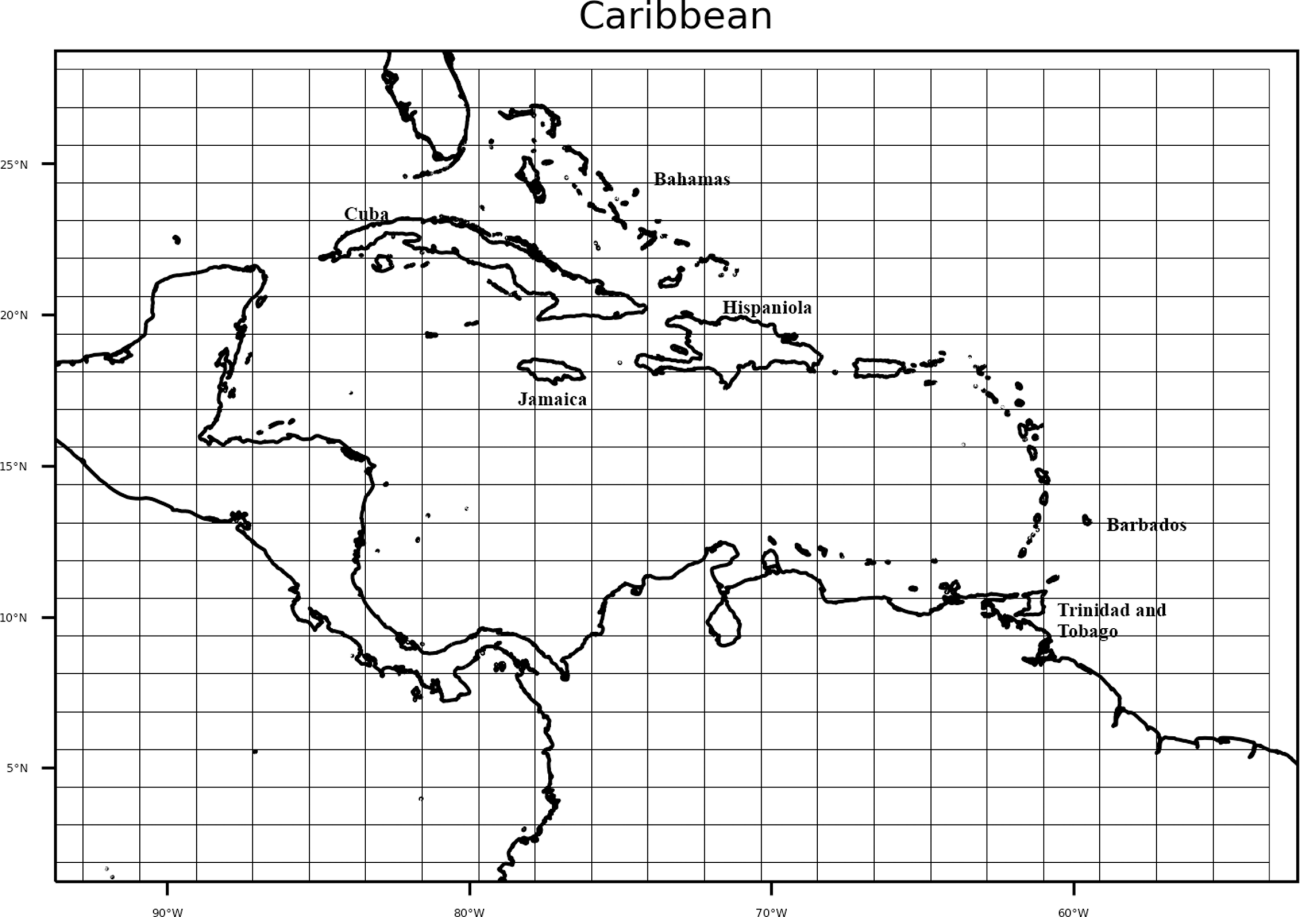
Study domain with model grid boxes overlaid.

The study domain is shown in Fig 1. It is bounded by latitudes 1.875°N to 25°N and longitudes 56.25°W to 90°W and encompasses the Bahamas in the north, Guyana in the south, Belize in the west, and the Caribbean Island chain. A similar domain is used in other climate-focused studies of the Caribbean [6,29].

### 2.2 Datasets

Data for SRM and non-SRM projections are derived from GeoMIP simulations using the Hadley Centre Global Environmental Model version 2-Earth System (HadGEM2-ES). Previous studies have shown that HadGEM2-ES under the Coupled Model Intercomparison Project Phase 5 (CMIP5) captures the climatology of the Caribbean reasonably well [6,29]. The model output is at a spatial resolution of 1.875^o^x1.25^o^ and is sourced from the Earth System Grid Federation (ESGF) repository [47]. Only the r1i1p1 realization is used as it is the only realization with all the input variables needed for the VRE models being employed at a daily temporal resolution.

The SRM data are based on HadGEM2-ES simulations using the G4, G4cdnc, and G4seasalt scenarios from the GeoMIP project [20]. The G4 scenario represents Stratospheric Aerosol Injection (SAI) SRM and is selected for its alignment with the goal of limiting global temperature rise to 1.5 ∘C  by the end of the century. The G4 scenario delays global exceedance of the 1.5 ∘C threshold by 33 years compared to the G3 scenario (8 years) relative to the attainment date under RCP4.5. To explore the impact of multiple SRM strategies in the Caribbean, the G4cdnc and G4seasalt scenarios representing Marine Cloud Brightening (MCB) are also examined.

Details of the SRM scenarios are as follows:

- G4: 5Tg of SO_2_ is injected annually into the lower stratosphere along the equator (16−25 km in altitude) [48] starting in 2020 [20].
- G4cdnc: Cloud droplet number concentrations are increased by 50% to force cooling by altering low-level clouds.
- G4seaSalt: 100 Tg/year of sea salt aerosols are sprayed into the marine boundary layer (30S-30N) below 680 hPa [49].

The G4seaSalt and G4cdnc simulations are excluded from the solar analysis due to the unavailability of irradiance data.

The non-SRM data is derived from HadGEM2-ES simulations using the RCP4.5 scenario. Among the four Representative Concentration Pathways (RCPs) characterizing future global GHG trajectories, RCP4.5 assumes GHG emissions peak around 2040 and stabilize with a radiative forcing of approximately $4.5\;{Wm}^{-2}$ after 2100. This trajectory leads to a mean global warming of about 2.5 ∘C above pre-industrial levels by the end of the current century [50]. RCP4.5 is selected because the SRM scenarios analyzed (e.g., G4) are premised on its emissions trajectory up to 2020/2021, i.e., prior to initiating aerosol injection [20]. Using RCP4.5 also facilitates comparison with findings from the first Caribbean SRM study [29].

Due to the limited availability of continuous long-term station observations for solar irradiance and wind speeds at specific heights in the Caribbean, reanalysis datasets are used to substitute for the observed historical period. The following datasets are employed:

- ERA5 Reanalysis: Produced by the European Centre for Medium-Range Weather Forecasts (ECMWF), ERA5 provides data on irradiance and wind with spatial resolution of 0.28125^⸰^x 0.28125^⸰^ [51,52]. It is considered robust and reliable for the Caribbean region [53].
- NCEP Reanalysis 2: Generated by the National Center for Environmental Prediction, this dataset has a courser resolution of 2.5^⸰^x 2.5^⸰^ and is widely used to represent regional wind regimes and circulation patterns [54,55].

These reanalysis datasets are used for validating the model simulations.

### 2.3 Methodology

The study methodology consisted of the four tasks detailed in sections 2.3.1 to 2.3.4.

#### 2.3.1 Simple model validation.

Notwithstanding its extensive use in the region, a simple validation of the HadGEM2-ES model is undertaken by comparing it with ERA5 and NCEP Reanalysis 2 data over the historical period of 1980–2005. This period is selected as it is the longest mutually shared historical period across the Reanalysis datasets and HadGEM2-ES under CMIP5. It is also similar to that analysed in the prior SRM Caribbean study [29], allowing for a comparison between results. Time series of global horizontal irradiance (GHI), 10m winds, and 925 mb winds are created by area averaging over the Caribbean domain. Mean annual and seasonal maps for the same variables are also generated for the domain and compared. The seasons used are December–January–February (DJF), May-June–July (MJJ), July–August (JA), and August–September–October–November (ASON). This is consistent with studies showing the Caribbean’s climatology as consisting of a main dry season (DJF), two rainfall seasons (MJJ and ASON), and a mid-summer dry period accompanied by low-level wind jet intensification (JA) [6,29]. The area-time averaged Root Mean Square Error (RMSE) and Mean Absolute Percentage Error (MAPE) values are calculated for each seasonal and annual map of irradiance and wind speeds, using the ERA5 and Reanalysis 2 datasets as reference.

#### 2.3.2 Assessment of SRM vs non-SRM future VRE resource potential.

The future changes in VRE resource potential under non-SRM conditions (RCP4.5) are first determined for two future 11-year periods, namely 2024−2034 and 2038−2048, with respect to a present-day historical period (1980−1990). The future periods correspond to years when the projected mean global surface temperatures reach 1.5 ∘C  and 2.0 ∘C  respectively above pre-industrial levels. The Caribbean SRM study [29] determined these periods by applying an 11-year moving average to a mean global surface temperature time series from 1850 onwards. The study determined that 2029 and 2043 (the midpoint of each 11-year future time-slice) were the first years when mean global surface temperatures reached and thereafter exceeded 1.5 °C and 2.0 °C for the HadGEM2-ES RCP 4.5 (non-SRM) simulation [29] for the r1i1p1 realization.

The future changes in VRE resource potential are calculated for the same 11-year periods for the SRM modelled scenarios (i.e., G4, G4cdnc and G4seasalt) relative to the RCP4.5 (non-SRM) results. In both cases (i.e., SRM and non-SRM plots), mean annual and seasonal composite maps are created for GHI and the height-specific wind magnitudes. Two-tailed p-values at the 95 % confidence interval (p≤0.05) are also calculated, and statistically significant areas marked with black hatching on each spatial plot.

#### 2.3.3 Assessment of SRM vs non-SRM future photovoltaic power generation.

The future solar PV energy production from spatially fixed solar technology is analyzed for the Caribbean using the Global Solar Energy Estimator (GSEE) package in Python [56]. Though concentrated solar power and two-axis tracking solar PV will likely be more heavily affected by SRM [45], the cost of fixed solar PV will likely continue to make it the more economical option for the Caribbean region in the near to mid-future. The GSEE package is used for its ability to directly ingest Global Climate Model (GCM) gridded data versus other open-source solar PV modelling packages which generally ingest data as a time series (e.g. PVlib, PVwatts).

GSEE requires composite maps of GHI, mean surface temperature (tas), and diffuse fraction as input. The RCP4.5 and G4 model datasets include the first two variables (referred to as rsds and tas respectively under ESGF). Daily values are extracted as it is the highest temporal resolution common to both datasets. The Boland-Ridley Lauret (BRL) model [57] in GSEE is used to estimate diffused fractions. It has been used with CMIP5 models with favourable results [58].

**Fig 2:**
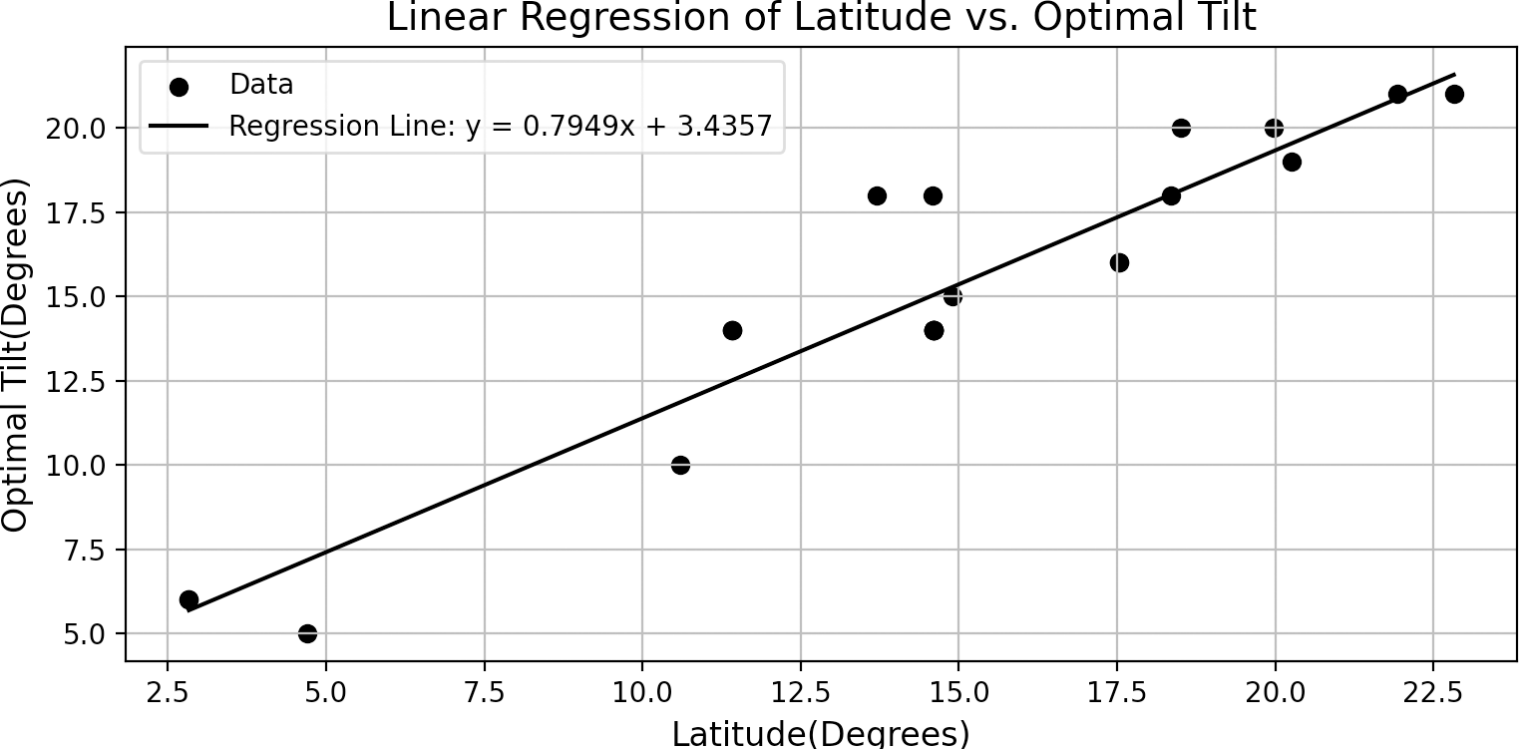
Linear Regression used for grid point filling of optimal tilt angles.


The optimal tilt angle at each grid point in the GSEE simulations is from Equation 1 which calculates tilt based on latitude. Equation 1 is derived using a linear regression method [56,58] and using estimated PVWatts results of optimal tilt angles for stations around the world [59]. The data for stations within the Caribbean domain are provided in Table 1 and the linear regression is shown in Fig 2.

**Table 1:** Latitude and Optimal tilt for stations within the Caribbean domain (Fig 1) used to determine the linear regression relationship for *Tilt* in equation 1. Table adapted from [59].

Country	Station Lat (⁰N)	Optimal Tilt (⁰)	Country	Station Lat (⁰N)	Optimal Tilt (⁰)
Antigua and Barbuda	14.60	14	Honduras	14.90	15
Bahamas	22.82	21	Jamaica	19.97	20
Barbados	14.60	14	Mexico	17.53	16
Belize	17.53	16	Nicaragua	11.42	14
Colombia	4.70	5	Panama	11.42	14
Costa-Rica	11.42	14	El Salvador	13.70	18
Cuba	21.93	21	Saint Kitts And Nevis	18.35	18
Dominica	14.60	14	Saint Lucia	14.60	14
Dominican Republic	18.50	20	St. Vincent/ Grenadines	14.60	14
Guyana	2.83	6	Suriname	2.83	6
Haiti	20.25	19	Trinidad and Tobago	14.60	14
Guatemala	14.58	18	Venezuela	10.60	10

Tilt=0.7949*lat+3.4357 (1)

The GSEE simulation is run using a 1000 W solar PV module arrangement per grid point with no-axis tracking (fixed) and an azimuth angle of 180∘. This ensures the plane of each solar PV module faces the equator which is south of the domain. The module type is crystalline silicon which is the default in GSEE. The panel type has a characteristic panel temperature coefficient of $0.035\;\frac{\circ \text{C}}{{Wm}^{-2}}$. The DC power output is calculated from the current-voltage (I-V) curve produced by using the single diode model and the power-irradiance relationship from the PVlib [60,61] package; see expressions (2 and 3).


$$I=I_{L}-I_{o}\left[e^{\left(\frac{V+IR_{s}}{nN_{s}V_{th}}\right)}-1\right]-\frac{V+IR_{s}}{R_{sh}}$$ (2)

Where $I_{L}$ is the light-generated current, $I_{o}$, is the diode reverse saturation current, $R_{s}$, is the series resistance, $R_{sh}$, is the shunt resistance, n is the diode ideality factor, $N_{s}$ is the number of cells in a series formation for a PV module, $V_{th}$ the thermal voltage and I and V are the module current and voltage respectively.


$$P=\frac{Gpoaeff}{1000}*P_{dc0}\left(1+\gamma_{P_{dc}}\left(T_{cell}-T_{ref}\right)\right)$$ (3)

DC power (P) Equation, where Gpoaeff, is the Irradiance to PV cells, $P_{dc0}$, is the Nameplate Rating, $\gamma_{P_{dc}}$ is the Temperature Coefficient of Power, $T_{cell}$, is the Cell Temperature, and $T_{ref},$ is the Reference Temperature.

The GSEE package assumes maximum power point tracking (MPPT). System losses outside of solar PV modules and Inverter losses are set to a 10 % reduction of power generation to account for AD-hoc sources of losses.

#### 2.3.4 Assessment of SRM vs non-SRM future wind farm power generation.

To the best of the authors’ knowledge, at the time of this study, there are no available open-source packages in Python that directly ingest gridded data (observed, reanalysis and/or model output) in NetCDF format for general-purpose wind power simulations, as GSEE does for PV simulations. Windpowerlib in Python ingests time series data [62]. Consequently, to perform gridded wind power simulations a two-stage process is used. The first stage utilizes bash shell scripting in combination with Climate Data Operators (CDO). The second stage involves ingesting the output of the bash shell in Python. In the first stage, the time series of each grid point of the GCM datasets for wind speed, pressure, and temperature at specified heights are extracted and transformed into individual CSV files. The CSV files for each variable per grid point are concatenated along with a generic CSV file of the same time series length containing a constant roughness length value. The formatting and labelling of the index columns are transformed according to the requirements of windpowerlib. At the end of the shell script, a 5-column CSV file is generated for each grid point, containing a time column, and four columns for the aforementioned variables. A moderate roughness length of 0.25 m is used since most of the domain is water [63].

In the second stage, each CSV file is passed through two windpowerlib wind turbine configurations. The first is the Enercon E126 Turbine, and the second is a Vestas V80 turbine. The latter simulation will enable future comparative studies as the Vestas V80 is the highest turbine used at the Wigton Wind Farm in Manchester, Jamaica, which is the largest wind farm in the English-speaking Caribbean [64]. The former simulation provides results for a common turbine hub height globally (100 m). The system details for each turbine are listed in **Table 2**.

**Table 2:** Windpowerlib wind turbine system specifications for the Enercon E126 and Vestas V80 turbines.

Turbine Specification	Enercon E126 Turbine	Vestas V80 Turbine
Rotor diameter (m)	127	80
Hub height (m)	99	67
Cut-in wind speed (${ms}^{-1}$)	2.5	4
Cut-out wind speed (${ms}^{-1}$)	25	25
Rated wind speed (${ms}^{-1}$)	14	15
Installed power capacity (MW)	4.2	2.0

**Fig 3:**
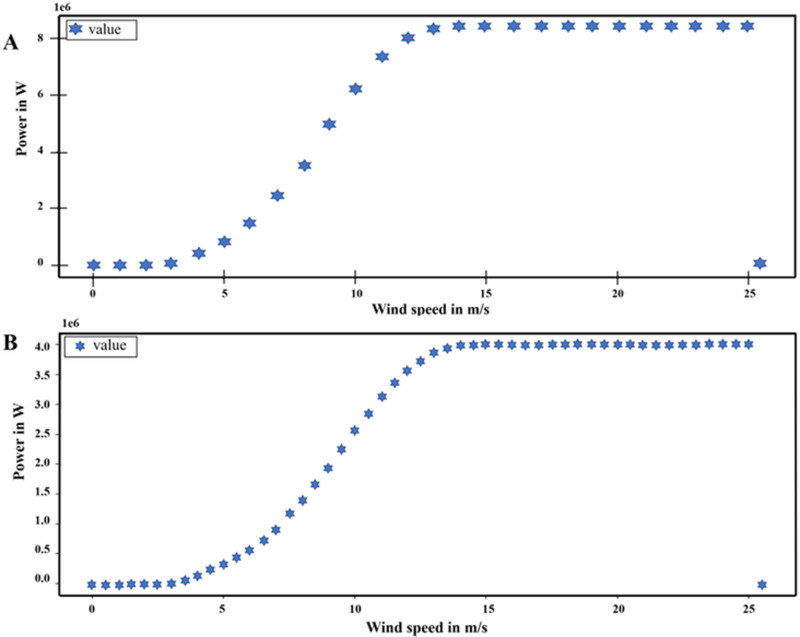
Wind Power Curves of Selected Wind Turbines in the windpowerlib package, A: Enercon E126 99 m hub height; B: Vestas V80 67 m hub height.

For both configurations, two turbines are set per grid point to simulate the wake losses of an aggregated power curve that comes with a wind farm setup. The aggregated power curves for each are shown in Fig 3 with an installed capacity of 8.4 MW and 4 MW respectively. For each simulation, any grid point with wind speeds below (above) the cut-in (cut-out) specifications of the turbines does not experience power generation.

The GCM datasets generally do not provide output at the hub heights of common wind turbines. Windpowerlib allows for the estimation of variable magnitudes at hub heights for turbine configurations using various equations [65]. The following equations are used:

I The log wind power profile law option with neutral stability conditions assumed for wind estimation:

$$v_{hub}=v_{mh}*\frac{\ln\left(\frac{h_{hub}-d}{z_{o}}\right)}{\ln\left(\frac{h_{mh}-d}{z_{o}}\right)}$$ (4)

where $v_{hub}$ is the wind speed at the selected turbine hub height, $v_{mh}$ and $h_{mh}$, the wind speed at the model height and the model height itself used respectively, $z_{o}$ the roughness length and d the zero-displacement height. The zero-displacement height is a fraction of the average height of above-ground level obstacles, where the produced turbulent flow at this fractional height dissipates wind speeds to zero. Given its mesoscale characteristic, this parameter is set to zero due to the low-resolution data afforded by the GCM.
II The ideal gas law option for density estimation:

$$\rho_{hub}=\frac{p_{hub}}{Rs*T_{hub}}$$

where

$$p_{hub}=p-\left(\left(h_{hub}-h_{mh}\right)*\frac{100}{8})\right)$$ (5)

where $p_{hub}$, $\rho_{hub}$ and $T_{hub}$ are the pressure, density, and temperature at the selected turbine height respectively, with Rs being the specific gas constant of dry air. The equation assumes a pressure gradient force of $-\frac{1}{8}\frac{hpa}{m}\;$, i.e., a decrease of 1 hPa in atmospheric pressure per 8 m increase in altitude [66].
III The default linear gradient option for temperature estimation:

$$T_{hub}=T_{mh}-0.0065*\left(h_{hub}-h_{mh}\right)$$ (6)

where $T_{mh}$ is the temperature at model height. The equation uses the International Civil Aviation Organization (ICAO) standard atmosphere assumption of a linear gradient of $-0.0065\;{Km}^{-1}$. This is a decrease of 0.0065 K in temperature for every 1 m increase in altitude to the top of the troposphere at 11 km [67].

The relationship between standard turbine power production and laminar wind flow is a cubic function given by:


$$P=\frac{1}{2}\rho Av^{3}c_{p}$$ (7)

Where P is the Power extracted from the wind, ρ the standard air density, A the rotor swept area, v the wind speed magnitude and $c_{p}$ the coefficient of performance.

## Section 3: Results

### 3.1 Climatological variation of solar and wind resource and simple model validation

**Fig 4:**
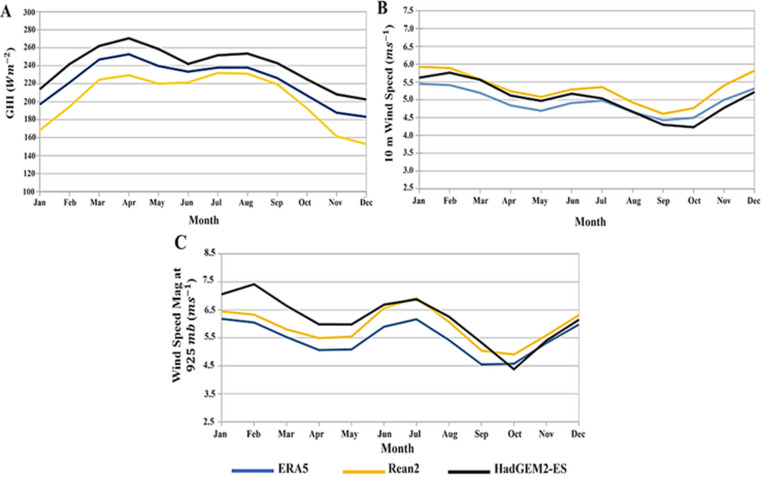
Monthly climatology plots for the Caribbean from the HadGEM2-ES (black), Reanalysis2 (yellow), ERA5(blue) datasets for GHI (A), 10m wind speed magnitude (B) and wind speed magnitude at 925mb (C). The average period is from 1980 to 2005*.*

Fig 4 presents the 1980–2005 climatology of GHI (A), wind speed at 10 m (B), and wind speed at 925mb/762m (C) averaged over the domain. Plots are shown for the two reanalysis datasets (ERA5 and Reanalysis2) and HadGEM2-ES simulation.

HadGEM2-ES overestimates GHI compared to the reanalysis products (Fig 4A). It, however, captures well the bi-modal pattern with peaks in April and July/August and a minimum in December. The results are comparable to a study for Cuba [68] that calculated the observed irradiance climatology at a station in Camagüey (21.422N, 77.850W, 122 m above sea level) using data from 1981–2016. Two peaks were found in April and July and a minimum in December [68].

The wind climatologies from ERA5 and Reanalysis 2 at 10 m (Fig 4B) and 925 mb/762 m (Fig 4C) are also reasonably captured by the model. HadGEM2-ES correctly simulates winter (January/February) and summer (July/august) peaks, as well as the minima in April/May and September/October [55]. This agrees with the Reanalysis assessments which have maxima in January and July. However, whereas HadGEM2-ES closely tracks the climatology of both Reanalysis datasets at 10 m for the entire year (Fig 4B), it slightly overestimates their wind speeds at 925 mb (Fig 4C) from January to May.

**Fig 5:**
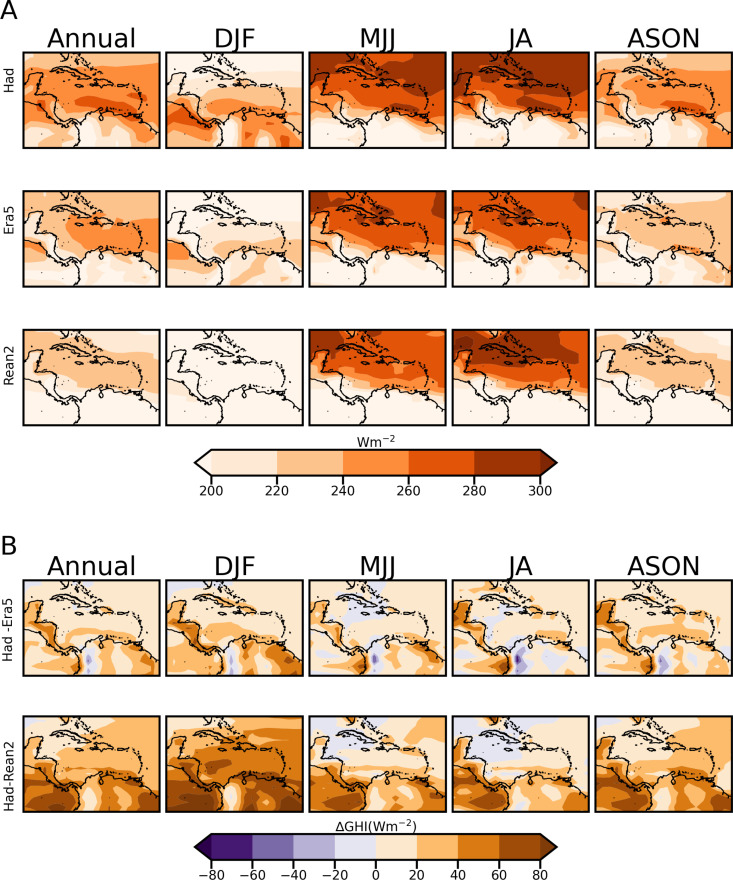
GHI annual and seasonal climatology plots of the Caribbean for the period 1980 to 2005. GHI plots are from HadGEM2-ES (row 1), ERA5 (row 2) and Reanalysis 2 (row 3) datasets (A) and the HadGEM2-ES relative to ERA5 and Reanalysis2 biases (B).

The mean annual and seasonal variation of GHI from the reanalysis datasets and HadGEM2-ES are plotted in Fig 5. Panel A gives the GHI magnitude and panel B shows the bias difference between the model and the two reanalysis datasets. Due to the daily resolution of the dataset, the plots of Fig 5 are not for peak sun-hours potential around 1000 Wm^-2^ (which is a standard metric common to solar potential) as they are skewed by night-time magnitudes. Notwithstanding, the solar resource potential for the entire domain annually and across seasons is shown. Table 3 summarises the RMSE and MAPE GHI performance of HadGEM2-ES relative to the Reanalysis datasets.

**Table 3:** Seasonal Root Mean Square Error (RMSE) and Mean Absolute Percentage error (MAPE) of GHI for HadGEM2-ES compared to ERA5 and Reanalysis 2.

Pairs	Metrics RMSE(Wm^-2^) MAPE (%)	Annual	DJF	MJJ	JA	ASON
Had relative to Rean2	**RMSE**	49.27	58.27	43.10	37.08	44.13
	**MAPE**	23.18	31.85	18.57	15.41	20.88
Had relative to ERA5	**RMSE**	29.78	30.27	29.92	27.26	28.62
	**MAPE**	11.27	12.47	10.79	9.64	11.24

The following are noted:

- Of the seasons presented, MJJ and JA provide the largest solar resource for the Caribbean (Fig 5A). This is seen in the reanalysis data and captured by HadGEM2-ES. HadGEM2-ES correctly simulates that the Greater Antilles and most of the Lesser Antilles have the highest annual and seasonal solar potential with respect to the rest of the domain. Over South America, it is the coastal and mountainous parts of Venezuela and Colombia, Guyana and western Suriname that experience the highest annual solar potential (see for example Fig 5B), while the south of Venezuela shows the lowest potential. Guyana and Suriname have their highest GHI in the ASON season.
- HadGEM2-ES overestimates the magnitude of mean GHI annually and seasonally (Fig 5B) notwithstanding its ability to reasonably model climatological variations in the spatial patterns (Fig 5A). There is generally a larger overestimation in the south versus the north of the domain. Small portions of the domain are underestimated including western Colombia and Cuba during MJJ and JA. Overestimation is smaller for ERA5, i.e., for the higher resolution reanalysis dataset. This is particularly evident in Table 3, where the RMSE and MAPE (less than 12.5% error) across all seasons are lower for ERA5 compared to Reanalysis 2. HadGEM2-ES performs best in capturing the months with the highest solar resource with MJJ and JA showing the lowest MAPE and RMSE of all seasons.

**Fig 6:**
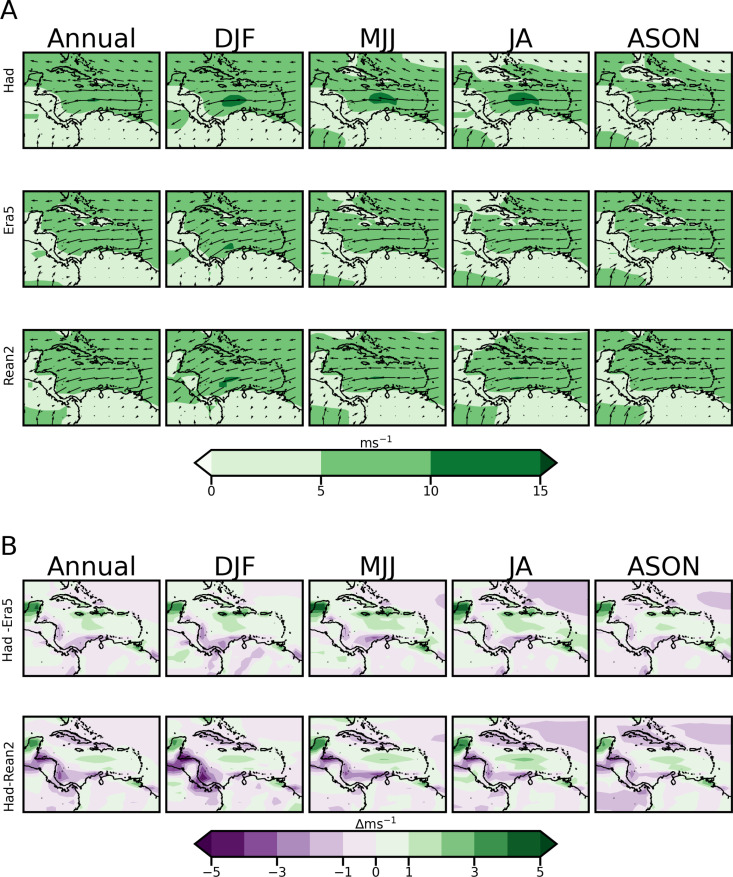
Annual and Seasonal climatology plots for wind speed magnitude with direction vectors at the A) $\begin{array}{c} 10\;m\; \end{array}$ AGL; Plots are from HadGEM2-ES (row 1), ERA5 (row 2) and Reanalysis 2 (row 3) datasets. B) Bias.

**Fig 7:**
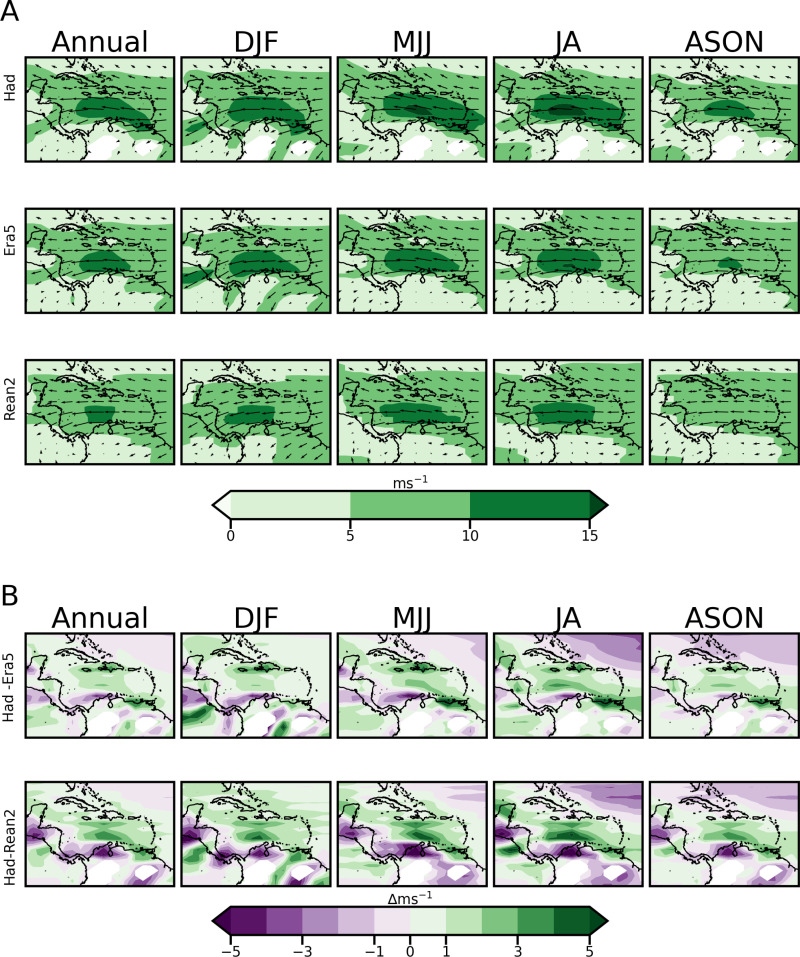
As in Figure 6 but for wind speed magnitude and direction at the $\begin{array}{c} 925\;mb\; \end{array}$ level.

The Caribbean has been identified as containing large wind power potential, serviced by the trade winds [55]. Fig 6A shows the corresponding mean annual and seasonal plots for 10-meter resultant wind speed magnitude, while 6B shows the bias differences. Fig 7A shows plots of wind speeds but at 925 mb to capture variations in the Caribbean Low-Level Jet (CLLJ) which is an important feature of the regional wind regime. The CLLJ is an intensification of easterly zonal wind observed in the lower troposphere (about 925mb) in the western Caribbean basin between 70°W–80°W and along 12–15°N [69,70]. It exhibits two maxima (minima) in February and July (May and October) [70]. Mean wind speeds during both peaks are generally greater than 12 m/s at the 925mb/hPa level, with the peak in July being the larger of the two. Fig 7B again shows bias differences. Table 4 summarises the RMSE and MAPE performance of HadGEM2-ES for wind speed at both 10 m/s and 925mb compared to the reanalysis datasets.

**Table 4:** Seasonal Root Mean Square Error (RMSE) and Mean Absolute Percentage error (MAPE) for 10m and 925mb wind speed for HadGEM2-ES relative to ERA5 and Reanalysis 2.

Variables	Pairs	Metrics RMSE(m/s) MAPE (%)	Annual	DJF	MJJ	JA	ASON
10m Wind Speed	**Had relative to Rean2**	**RMSE**	1.54	1.65	1.47	1.47	1.51
		**MAPE**	26.16	26.25	25.41	26.07	27.10
	**Had relative to ERA5**	**RMSE**	1.38	1.40	1.42	1.43	1.33
		**MAPE**	25.67	25.35	26.04	27.00	25.93
925mb Wind Speed	**Pairs**	**Metrics**	**Annual**	**DJF**	**MJJ**	**JA**	**ASON**
	**Had relative to Rean2**	**RMSE**	2.49	2.66	2.49	2.50	2.31
		**MAPE**	51.97	56.73	47.03	44.30	46.95
	**Had relative to ERA5**	**RMSE**	2.37	2.56	2.29	2.27	2.18
		**MAPE**	63.41	68.96	56.36	57.28	57.01

The ERA5 plots (Fig 6A) show that wind power potential at 10 m for large parts of the domain, especially in the Caribbean Sea, falls within the common cut-in wind speed of commercial wind farms (~3m/s). Most of the Caribbean islands have average speeds of 5m/s and above. HadGEM2-ES reasonably replicates the prevailing large-scale wind magnitude patterns at 10 m including wind direction shifts during the year. The bias in Fig 6B reveals that the model both underestimates and overestimates the 10m wind speed compared to the two reanalysis datasets, without a clear dominant trend in performance. The largest underestimations occur over Central America relative to Reanalysis 2, while overestimations are observed over Jamaica and Hispaniola relative to ERA5 and over the Yucatan Peninsula relative to both reanalysis datasets. Table 4 shows that the model performs slightly better against ERA5 than Reanalysis 2 for 10 m winds, with the lowest RMSE and MAPE observed during DJF and ASON.

In Fig 7A, HadGEM2-ES reasonably captures the positioning of the CLLJ and its bi-annual intensification when compared to the reanalysis datasets. The DJF, MJJ and JA plots show larger wind speeds in the CLLJ region than ASON at 925mb. HadGEM2-ES, however, overestimates the spread of the jet at this level, in addition to producing a July peak at the 10m level which is not as pronounced in the reanalysis datasets. The bias in 925mb in wind, shown in Fig 7B, indicates that the largest underestimations relative to Reanalysis 2 now extend to parts of South America, in addition to the regions identified for 10m wind in Fig 6B. Overestimations are observed over the CLLJ region compared to both reanalysis datasets. Although the model provides a reasonable spatial representation of the observed patterns, Table 4 shows percentage errors $\begin{array}{c} >40\%\; \end{array}$ for RMSE and MAPE values for 925mb winds compared to both reanalysis datasets. This overestimation must be considered for wind power simulations at altitudes above 10m if wind speeds from HadGEM2-ES are directly used. The statistical results support the choice to use the estimation functionality of windpowerlib instead of the interpolation functionality as outlined in section 2.3.4.

### 3.2 VRE resource change due to SRM application

**Fig 8:**
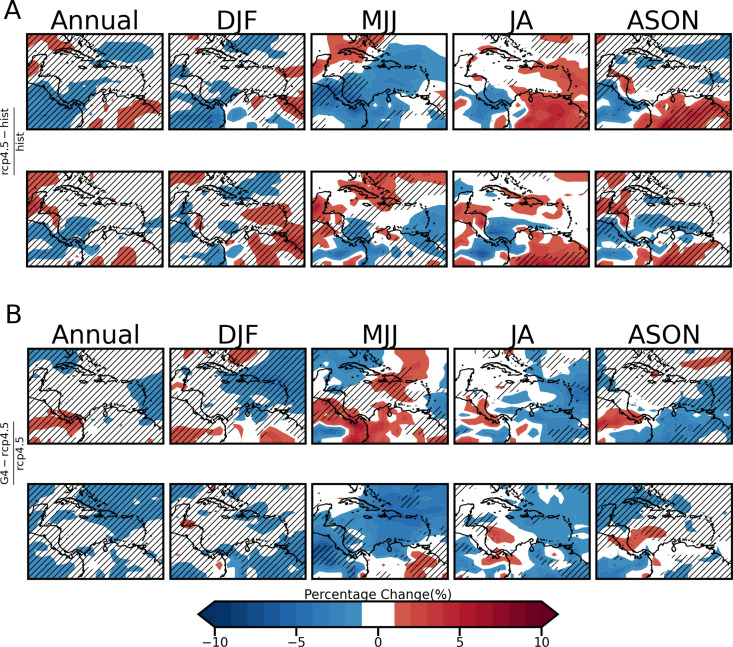
Percentage change maps for A) RCP4.5 relative to historical and B) G4 with respect to RCP4.5 for GHI. Changes are between composites derived for the 11-year periods centered on when RCP4.5 indicates global average temperatures attain the 1.5 ∘C  and 2.0 ∘C thresholds. Units are in percent and statistically significant changes are hatched at the 95% confidence level.

**Fig 9:**
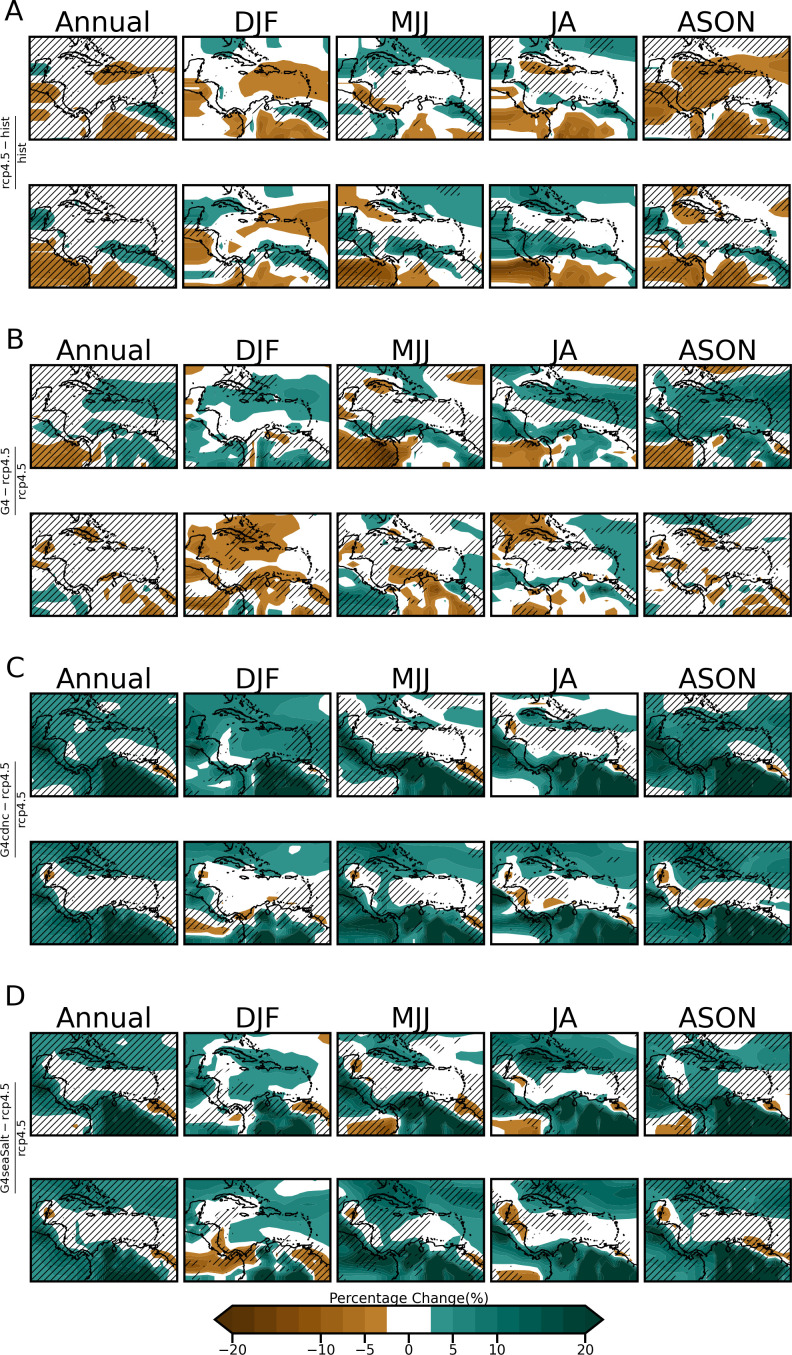
Percentage change maps for A) RCP4.5 relative to historical and B) G4, C) G4cdnc and D) G4seaSalt with respect to RCP4.5 for 10m wind speed. Changes are between composites derived for the 11-year periods centered on when RCP4.5 indicates global average temperatures attain the 1.5 and 2.0 thresholds. Units are in percent and statistically significant changes are hatched at the 95 % confidence level.

Panels A of both Figs 8 and 9 show respectively the percentage differences in irradiance and 10 m wind speed under RCP4.5 (no SRM) for the two future time slices corresponding to the 1.5 ∘C  and 2.0 ∘C global temperature thresholds (hereafter Δ1.5 ∘C  and Δ2.0 ∘C ) relative to a present-day historical period. As previously noted, the future time slices are 2024–2034 and 2038–2048. Panel A maps are referred to as the ‘climate change’ plots as they indicate changes in the VRE resources due to continued increases in GHG emissions following the RCP4.5 pathway but with no SRM applied. Panels B of the same figures show the percentage difference for the same Δ1.5 ∘C  and Δ2.0 ∘C years but under G4 (for GHI) and G4, G4cdnc and G4seaSalt (for wind speeds) relative to RCP4.5. Panel B therefore represents the further change from panel A due to SRM delaying the attainment of 1.5 ∘C   and 2.0 ∘C to later in the century.

In Fig 8 the following are noted about the climate change plots (panel A):

- In the figures changes between −1 to 1 % are considered negligible and unshaded. At Δ1.5 ∘C , there is negligible change in annual mean irradiance over most of the Caribbean islands except in the northwest Caribbean and specifically over western Cuba (increase), Haiti and the Turks and Caicos (decrease). The latter changes do not surpass ±6 %. There are similar magnitude changes in other parts of the domain, including the Pacific coast of Central America and Panama (increase) and northeastern South America (decrease). The summer seasons show the widest spatial changes in irradiance across much of the domain. In MJJ, most of the Caribbean (except western Cuba) experiences a decrease in irradiance, which is reversed in JA. The changes in these seasons do not surpass ±8 % and are statistically insignificant across most of the domain. For DJF and ASON most of the changes occur over the portion of the Central and South American landmasses captured in the domain.
- At Δ2.0 ∘C , negligible change is noted for the annual mean irradiance over the Caribbean islands, with no dominant direction of change for the seasons plotted. At Δ1.5 ∘C , the summer seasons exhibit the highest changes, which still do not surpass ±8 %. The tendency is for an increase in irradiance over the Greater Antilles in summer. Changes in DJF are between −5 and 7% and between −5 to 6 % in ASON. There is a notable reversal of the trend during MJJ for Hispaniola at Δ1.5 ^o^C (decrease) versus Δ2.0 ∘C (increase). Regions exhibiting statistically significant change are roughly the same for both future periods.

The following are noted for the G4 plots of Fig 8 (panel B):

- The SRM influence on the Caribbean under G4 for the Δ1.5 ∘C   years is a further −1 to −3% reduction in mean annual solar potential over the Lesser Antilles and western Cuba. A 2–4% increase is seen across Panama. The decrease over western Cuba counters the small increase relative to the present seen in the same region under the non-SRM simulation (panel A). There is a similar reversal of trends seen over the Caribbean islands from Hispaniola eastward during MJJ (small decrease under non-SRM to small increase under SRM) and JA.
- For the Δ2.0 ∘C  years, the area of decrease expands and there is no part of the domain where a mean annual increase in GHI occurs, compared to negligible change under RCP4.5. The annual pattern shows a −1 to −3% reduction in solar potential across most of the Bahamas, the central to the eastern portion of Cuba, the entirety of Hispaniola and Puerto Rico, and a small portion of the Northern lesser Antilles. The 8–10% increase in GHI for Δ1.5^o^C years during MJJ for the Pacific coast of Panama, the west coast of Colombia and the islands of Hispaniola and Puerto Rico, as well as the 3–5% increase over Colombia and Venezuela, are reversed during the Δ2.0 ∘C years for the same season, with reductions of −1 to −5% seen. During DJF and ASON changes are statistically significant across almost the entire domain.

In Fig 9, −2.5 to 2.5% is considered a negligible change and is unshaded.

The following are noted about the climate change plots (panel A):

- Except for decreases over Hispaniola and Jamaica, there is negligible change in mean annual 10 m wind speed over the Caribbean at Δ1.5 ∘C. Seasonally, DJF and ASON exhibit the widest spatial spread of changes characterized by reductions over parts of the Greater and Lesser Antilles (except western Cuba in DJF), Central America, and South America (except its northern coastline). The changes across these two seasons range from −13–17%. Only the changes in ASON are statistically significant. Changes over the Caribbean islands are negligible during the summer seasons of MJJ (except western Cuba) and JA, and elsewhere in the domain do not surpass ±9 %.
- At Δ2.0 ∘C , the annual mean change in wind speeds for the Caribbean islands is again negligible. Only DJF exhibits reductions over the Caribbean (also seen at Δ1.5 ∘C ), however, the decreases previously noted during ASON at Δ1.5 ∘C  are now limited to the west of Jamaica. Changes for these seasons are less than 12 %. The northern coastline of South America consistently exhibits increases in wind speed across all seasons with the summer seasons exhibiting the widest spatial spread. Changes reach up to +18 %. No notable reversals over land between the two global temperature thresholds are seen.

Whereas there is generally a similarity in the difference patterns induced by G4cdnc and G4seaSalt (panels C and D) at both global temperature threshold periods, there are notable differences for G4 (panel B) especially for the later time slice. This may suggest a distinction in impact dependent on choice of SRM methodology.

The following are noted from the G4 plots (B):

- During Δ1.5 ∘C  years, there is an increase in mean annual wind speed over most of the Greater Antilles. Increases are also seen across most seasons, except MJJ when the region of increase is only over eastern Cuba, Hispaniola and the Bahamas. This coincides with the region where [29] notes a drying effect under SRM (Fig 9 of that study). ASON has the largest spatial spread of increased wind resources ranging from 2.5 to 15 %, with changes across the entire domain being statistically significant. This counters the reductions previously noted under climate change in these regions.
- There is negligible further change in mean annual wind speeds across the domain for Δ2.0 ∘C   years under G4. This is also true for ASON which at Δ1.5 ∘C saw significant increases in wind speed over the Caribbean. There are reductions (between 10 and 20%) over the northern coastline of South America in DJF, reversing increasing trends seen under the non-SRM scenario. In MJJ a similar reversal is seen but restricted to the northwest coastline. Interestingly, it is also noted that for the Pacific coast of Panama, an increase (decrease) in wind speed is seen for Δ2.0 ∘C  (Δ1.5 ∘C ) years during MJJ. This is opposite to the trend observed for GHI for coastal Panama, i.e., decreased (increased) GHI for Δ2.0 ∘C (Δ1.5 ∘C ) years (Fig 8). We highlight this to note the implications for planning for VRE resource utilization under SRM. For example, during MJJ which is usually one of the energy-intense seasons of the region, offshore solar could be implemented for Panama during the 1.5 ∘C  period to take advantage of the increased solar resource, and offshore wind implemented in the 2.0 ∘C  period to cover the anticipated solar reduction shortfall.

The following are noted from the G4cdnc and G4seaSalt plots:

- G4cdnc and G4seaSalt induce increases in the mean annual wind over the majority of the domain for Δ1.5 ∘C  and Δ2.0 ∘C  years. The exception is the southern islands in the Lesser Antilles. The highest increases are, interestingly, not found in the vicinity of the CLLJ but across most of South and Central America, with increases ranging from 10% to more than 20%.
- Very few land areas see a reduction in wind speeds for both G4cdnc and G4seasSalt. The most prominent reductions are found in DJF for G4seaSalt for Δ2.0 ∘C  years over Panama, Guyana and Suriname and range from 2.5 to 10%.

### 3.3 *Impact of SRM on photovoltaic power output change at 1000W installed capacity per grid box for* Δ1.5^o^C and Δ2.0^o^C

**Fig 10:**
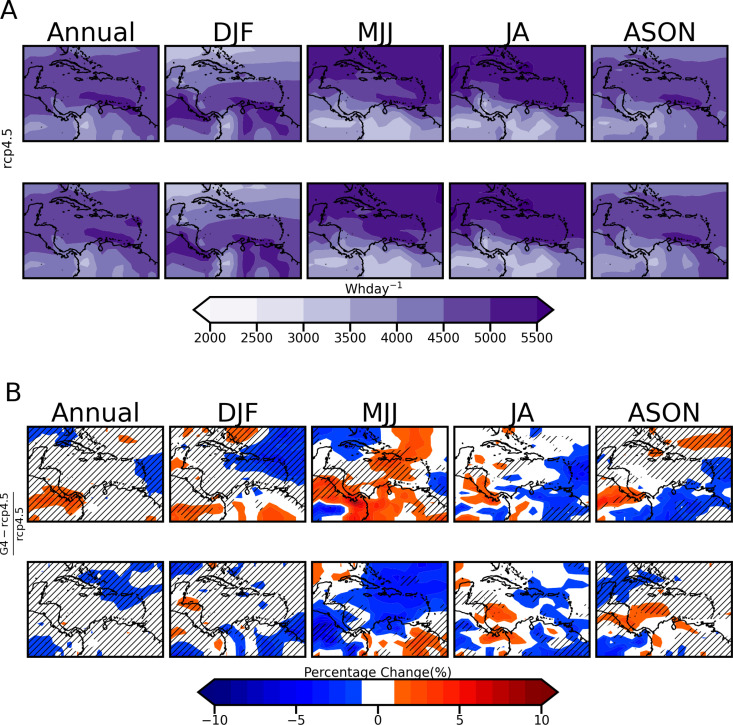
11 Year composites for A) Photovoltaic Energy Generation under RCP4.5 and B) Energy Generation Percent change between G4 and RCP4.5.

Fig 10A shows annual and seasonal future photovoltaic energy generation in the Caribbean for Δ1.5 ∘C  and Δ2.0 ∘C  years under RCP4.5. The percentage difference for the same years when SRM (G4) is applied is shown in Fig 10B. Table 5 shows the spatially averaged seasonal and annual percentage change, with the highest increase under the 1.5 ∘C  world and the highest decrease in the $2.0^{\circ }\text{C }$world for G4 in comparison to RCP4.5. The following are noted:

**Table 5:** Spatial seasonal averages of Solar PV energy generation percent changes of G4 relative to RCP4.5.

Warming Threshold (^o^C)	Annual (%)	DJF (%)	MJJ (%)	JA (%)	ASON (%)
1.5	−0.02	−0.25	0.59	−0.47	−0.29
2	−0.52	−0.50	−1.37	−0.42	−0.18

- MJJ and JA seasons show the greatest power production. This is expected from the resource potential maps of Fig 5. Production from $4.5\;5.5\;\frac{kWh}{day\;}$ for a 1 kW  solar PV module per grid point is obtained across the Greater Antilles (Fig 10A).
- The plot of percentage energy difference mirrors the percentage irradiance difference shown in Fig 8 for both Δ1.5^o^C and Δ2.0^o^C. This is also evident in [45]. Across the domain for both Δ1.5 ∘C and Δ2.0 ∘C  years, SRM does not induce a change in power production beyond 10% at the GCM scale.
- For both Δ1.5 ∘C  and Δ2.0 ∘C  years for the annual plot, the portion of the domain that falls within the negligible range (1 to −1%) is greater compared to the solar potential analysis, especially for Δ2.0 ∘C. This suggests the possibility of a slight improvement in solar PV efficiency for some grid points due to reductions in ambient temperature driven by SRM. This would potentially negate any small energy generation reductions from reduced irradiance.

### 3.4 Impact of SRM on 67 & 99m turbine wind power output at 8.4MW and 4MW installed capacities respectively per grid box for Δ1.5 ∘C  and Δ2.0 ∘C

**Fig 11:**
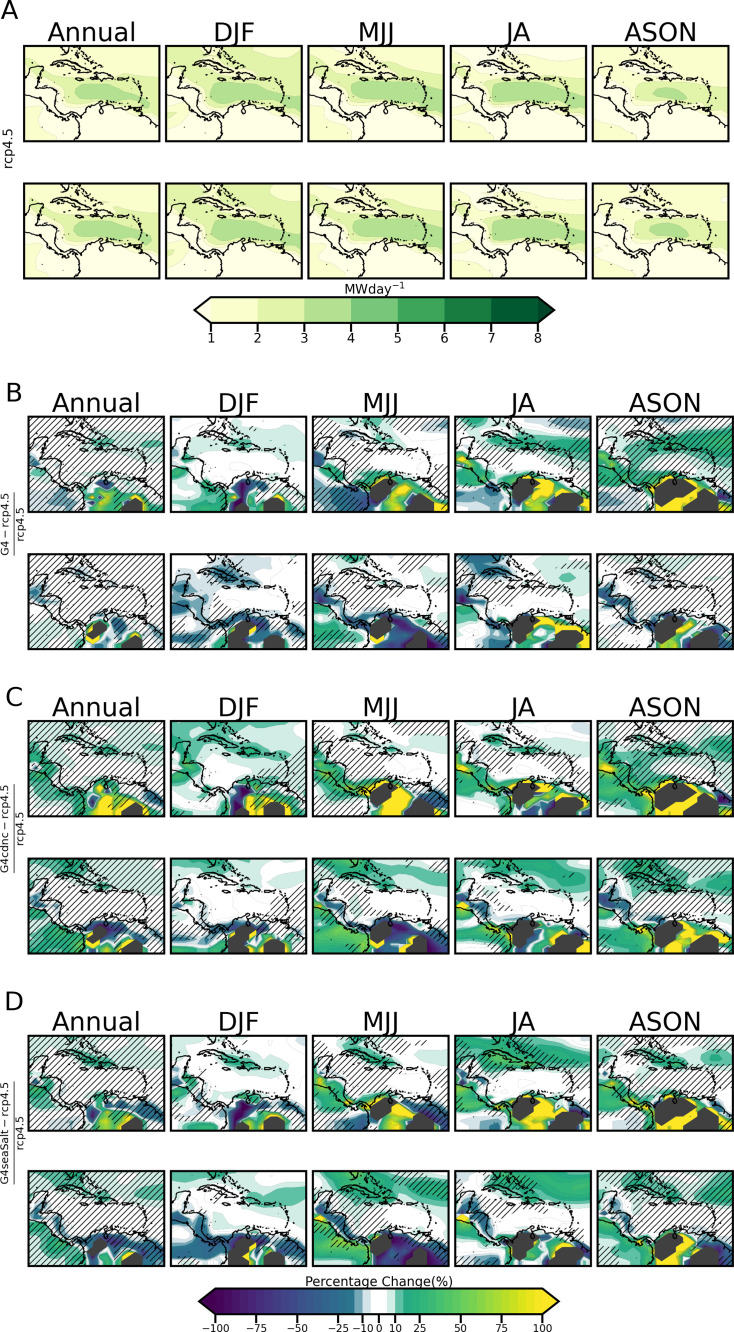
11-Year Composites for A) the Power Generation of a 67m hub height turbine under RCP4.5 and the Power Generation difference between B) G4, C) G4cdnc and D) G4seaSalt with respect to RCP4.5. The grid points where there is no power generation under RCP4.5 but generation when SRM is employed is masked with a grey shade.

Fig 11A shows the annual and seasonal power production of the Vestas V80 turbine under RCP4.5. As expected, the CLLJ region which is the most energy-dense area in the region [55] experiences the highest power production, with slight difference between Δ1.5 ∘C and Δ2.0 ∘C . Wind speeds up to $14-16\;{ms}^{-1\;}\;$ are attained which are consistent with the rated wind speed of the turbine of $15\;{ms}^{-1}$. Consequently, the rated power of 4 MW is attained.

Fig 11B, 11C and 11D shows the SRM-induced percentage change for Δ1.5^o^C and Δ2.0^o^C years for G4, G4cdnc and G4seaSalt respectively. In these plots −5% to 5% is considered the negligible range.

For Δ1.5 ∘C  years the following are noted:

- Most of the Greater Antilles and the northern Lesser Antilles experience a 5–15% increase in annual power production/generation under G4. In contrast, the western end of Cuba, the southern portion of the Lesser Antilles, the central Caribbean, most of Central America and the Northern cluster of Bahamian islands show negligible change (less than 5%). The largest power increases occur over South America, with southern Colombia, Venezuela, Guyana and Suriname experiencing increases between 50 and 100% for MJJ, JA and ASON. Guatemala and a small portion of Panama/border of Colombia achieved power increases of 100% and above during the JA and ASON respectively under G4. ASON shows the greatest spatial spread in increases for G4.
- Power reductions under G4 are most prominent over Colombia in DJF and off the coast of Panama in MJJ. The reductions are on the order of 5–20%.
- Under G4cdnc most of the domain exhibits overland mean annual power increases within the range of 10–100%. The parts of Central America that exhibited little mean annual change under G4 now see power gains of between 10 and 50%.
- The 10–20% reduction in annual power production seen over Guyana and Suriname under G4cdnc is slightly expanded under G4seaSalt.

For the Δ2.0 ∘C  years the following are noted:

- The majority of the Greater and Lesser Antilles do not experience significant changes in annual power generation under G4. The exception is central Cuba and Hispaniola which experience a 5–10% reduction. For Hispaniola, this is a reversal of the increase seen at Δ1.5 ∘C , which is also seen in DJF in a more pronounced way with decreases from 5–20%
- MJJ plots display the largest seasonal spatial spread of reductions situated over South America for G4, G4cdnc and G4seaSalt, with the highest reductions ranging from 50 to 100 %.
- MJJ and JA exhibit the largest power increases under both G4cdnc and G4seaSalt over the Greater Antilles, with parts of Cuba and the Bahamas experiencing increases between 25 and 75%. Changes in JA are important as the summer months usually coincide with the highest energy consumption due to the high cooling demand. Fluctuations in wind power during summer would challenge the generator’s ability to meet demand when it is most needed.

Table 6 presents spatial averages for each season. The highest percentage increases occur in DJF under G4cdnc at 1.5 ∘C  (64.72%), followed by MJJ under G4cdnc at 1.5 ∘C  (53.17%). Only ASON under G4 at 2.0 °C experiences a reduction (−0.56%). It is noted that under all SRM scenarios and for both Δ1.5 ∘C  and Δ2.0 ∘C years there are some grid points over south and central America which do not achieve power generation under RCP4.5 (i.e., average wind speeds are less than the cut-in wind speed of the Vestas V80). This is reversed under SRM. The implication is that SRM induces the increase needed for these grid points. These points are masked with a grey shade in the plots to differentiate them from the other power increases of SRM relative to RCP4.5.

**Table 6:** Spatial seasonal averages of Vestas 67m turbine power generation percent changes of G4, G4cdnc and G4seaSalt relative to RCP4.5.

SRM scenario	Warming Threshold (^o^C)	Annual (%)	DJF (%)	MJJ (%)	JA (%)	ASON (%)
G4	**1.5**	4.86	4.44	9.58	31.95	33.57
	**2**	8.07	39.56	2.31	29.48	−0.56
G4cdnc	**1.5**	16.71	14.78	18.74	29.70	40.80
	**2**	50.03	64.72	53.17	19.51	19.38
G4seaSalt	**1.5**	5.36	3.00	9.14	31.22	27.24
	**2**	2.84	33.04	3.31	14.69	12.78

**Fig 12:**
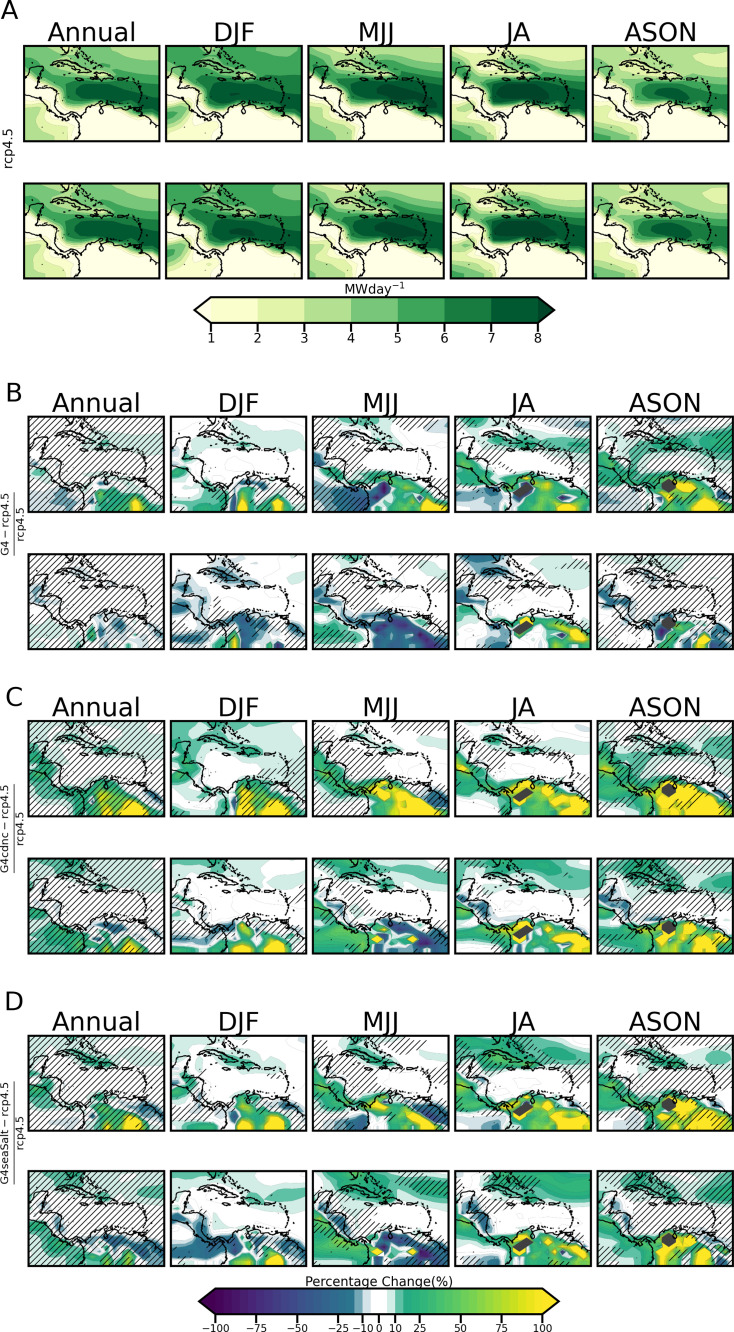
Same as Fig 11 but for the Power Generation of a $\begin{array}{c} 99\;m \end{array}$ hub height turbine.

Fig 12A shows the annual and seasonal power production of the Enercon E-126 turbine under RCP4.5 for Δ1.5 ∘C  and Δ2.0 ∘C years. For Fig 12, −5% to 5% is considered negligible. The DJF season achieves the greatest spread of highest power production yields ranging from 4−6 MW in the Greater Antilles and from 6 MW  to the installed capacity of 8 MW  on average for the Lesser Antilles and over the CLLJ focal area. For both Δ1.5 ∘C  and Δ2.0 ∘C, Venezuela, Guyana, Suriname (western), Central America and Colombia achieve the lowest power production for the region, always ranging from 0.5− 2 MW  (except the Yucatan Peninsula which produces 5−6 MW). This correlates to the lower wind speeds seen for HadGEM2-ES in Central America and South America in Fig 6A. Table 7 presents the seasonal spatially averaged percentage changes across the region.

**Table 7:** Spatial seasonal averages of Enercon 99m turbine power generation percent changes of G4, G4cdnc and G4seaSalt relative to RCP4.5.

SRM scenario	Warming Threshold	Annual (%)	DJF (%)	MJJ (%)	JA (%)	ASON (%)
G4	**1.5**	4.42	7.87	4.07	10.56	77.26
	**2**	−0.52	−4.11	−4.08	6.46	3.74
G4cdnc	**1.5**	18.60	36.03	25.88	23.21	89.75
	**2**	8.64	26.89	7.79	20.80	29.15
G4seasalt	**1.5**	8.66	14.53	12.09	25.20	84.23
	**2**	4.44	4.13	7.49	14.77	24.21

Fig 12B, C and D show the percentage change in power production under the SRM scenarios. As expected, in comparison to Fig 11B, C and D, the number of grid points that are masked grey are reduced at the 99 m  height. This is due to the greater hub height to roughness length ratio $\frac{h_{hub}}{z_{0}}$ yielding a higher scaling parameter for wind speed extrapolation. More points under RCP4.5 therefore now obtain speeds to generate power from the turbine in conjunction with the lower cut-in wind speed of the turbine. SRM at these points therefore no longer provisions cut-in wind speeds but instead provides an increase or decrease in power production. It is also noted that increases over South America of between 75 and 100% are more prominent under G4cdnc and G4seaSalt than under G4. Table 7 shows that the highest increases occur in ASON under G4cdnc at 1.5 ∘C (+9.75%), followed by ASON under G4seaSalt at 1.5 ∘C (84.23%). Table 7 indicates that the largest reductions occur in DJF under G4 at 2.0 ∘C (−4.11%), followed by MJJ under G4 at 2°C (−4.08%).

**Fig 13:**
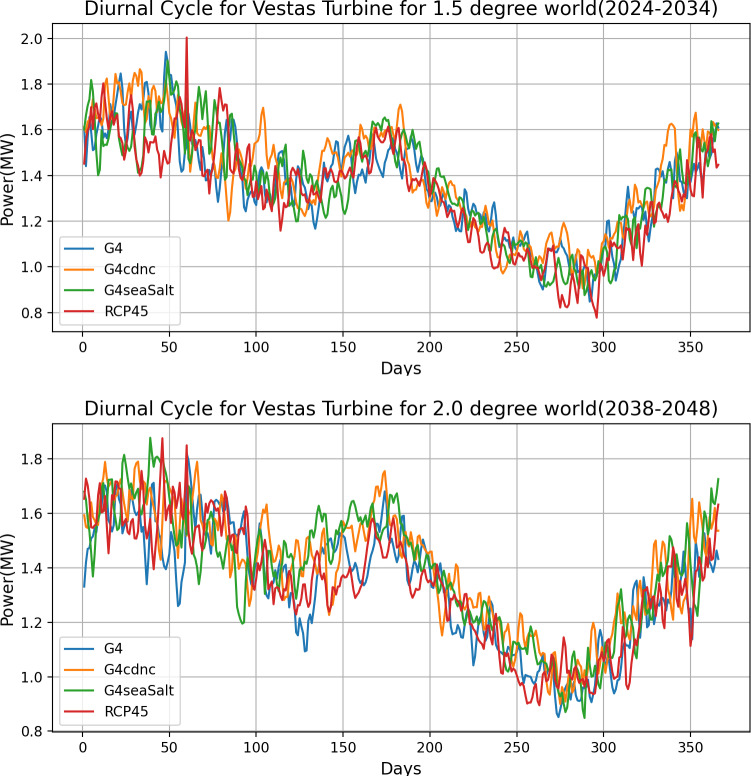
Diurnal Cycle of G4, G4cdnc, G4seaSalt and RCP4.5 for A) a $\begin{array}{c} 1.5\;\circ \end{array}$ world and B) $\begin{array}{c} 2.0\;\circ \end{array}$ world for the $\begin{array}{c} 67\;m \end{array}$ turbine.

With the significantly varied results of wind power potential changes under SRM, the extent of the effect of these changes is further examined in Fig 13. The plot shows the diurnal cycle for power production for each SRM scenario and RCP4.5 for the 1.5 ∘C  and 2.0 ∘C world. The diurnal cycle for the 67 m turbine is not shifted by any of the SRM methods compared to RCP4.5. This suggests that SRM does not alter the instances of occurrence of low and high-power production but has the potential to notably change the value of expected power output.

## Section 4: Discussion

Historical events, such as the 1991 Mount Pinatubo eruption, demonstrate the potential of SRM to induce planetary cooling and threaten VRE-facilitated energy security. For example, concentrated solar producers in California, USA experienced a 34% reduction in generation due to decreased direct radiation a year after the eruption [13].

In this study, solar PV and wind power simulations are used to identify changes in VRE potential in the Caribbean if SRM techniques are globally employed to delay achieving global warming thresholds of 1.5 ∘C  and 2.0 ∘C. VRE technologies are seen as key to achieving energy security in the Caribbean. They allow utilities to scale localized energy generation at a cheaper rate to meet the increase in a country’s energy demand that comes with development. The localization of generation fosters energy independence and accommodates the development of energy-intense product-based industries at a relatively lower cost than imported energy. Such industries have fuelled the sustainability of the economies of scale of many developed countries but have been almost unattainable to the same degree for many SIDS.

The results of this study are framed within the temperature thresholds of the Paris Agreement, in contrast to the approaches in [45,46]. This provides a policy-driven framework for classifying the possible impacts of an unknown such as SRM within a national energy plan. Additionally, applying power simulation methodologies across the entire domain, without excluding historically low VRE-resource areas, helps Caribbean energy planners communicate with regional governments about how much of a factor of change SRM might be in influencing future energy resources. If regions with historically low resources show signs of viability under SRM-induced climate conditions, this could significantly affect future planning.

While this study offers several contributions to the existing literature [45,46], one major limitation is the temporal resolution of the model data used. Unlike [46], which employed a model with hourly output for a more granular PV assessment, this study uses the HadGEM2-ES model, whose highest mutually available resolution across all scenarios is daily for all variables. Consequently, the PV assessment aligns more closely with [45]. The PV reductions observed in the Caribbean in this study are of similar magnitudes to those reported in [45].

It is found that the mean annual future solar resource for the Caribbean islands is not significantly altered at 1.5 ∘C  and 2.0 ∘C  under RCP4.5. There is also no major alteration in the mean annual irradiance or PV power generation due to the introduction of SRM. For example, no changes in PV generation (increases or decreases) are seen in excess of 10%. The solar PV results obtained for the Caribbean are consistent with the results of the global PV study at the GCM level under SSI, which also found small changes in production [45].

In contrast, the future wind resource and power analysis shows sizable changes due to the introduction of SRM. Changes of between 5 and 20% in wind resources occur across most of the domain, with some sections of Central and South America showing increases in excess of 20%. For wind power generation, changes predominantly reflect increases which are up to 75% in the Greater Antilles and exceed 100% in Central and South America. This is generally true for both global warming thresholds. There is however some dependence on the choice of SRM method, with G4 (stratospheric SO_2_ injection) producing smaller wind speed increases. Applying SRM results in major increases to parts of the domain that have the lowest power generation under the non-SRM scenario and negligibly influences the parts with the highest power generation under the non-SRM scenario such as the central Caribbean.

The implication is that, whereas the use of SRM may delay attaining the thresholds [29], it will also significantly change the regional VRE landscape, by potentially providing predominantly more wind power resources, especially under MCB SRM. This perceived benefit must be weighed against the other impacts stronger winds would bring to the region (e.g., greater potential for inland flooding when coupled with higher projected sea levels); as well as the other changes in climate under SRM, especially the progressively drier Caribbean which emerges [29].^.^ Given the growing underperformance of nations in meeting climate goals and the increasing likelihood of global players considering SRM, Caribbean energy planners can use the methodology used in this study as a starting point to develop compensatory measures within their fiscal frameworks in the event of SRM deployment. However, since the outcomes are closely tied to the specific turbine types used, the results can only be directly incorporated into energy security plans if the same turbine models are adopted by energy planners.

Future studies should shift away from a regional raw power potential and generation context to that of a fine-scale national-level temporal assessment of the interaction of SRM-induced PV generation with the energy demand profile of a nation. The results of the spatial assessment of SRM on wind resources and wind power production in the Caribbean also suggest that finer-scale studies of the wind assessment would provide added value to characterizing SRM’s impact, e.g., over the smaller islands of the Lesser Antilles. Such finer-scale studies would also permit the assessment of changes in the underlying mesoscale dynamics of the wind regime under SRM. Future work will include applying dynamical downscaling thereby allowing for nation-specific results. This paper also adds to the global catalogue of impacts-based SRM studies but is a first of its kind for the Caribbean.

## 5 Conclusion

The possible effects on VRE resources in the Caribbean of using SRM to limit future global temperature rise are explored. Three SRM scenarios, namely, G4, G4cdnc and G4seaSalt are considered to provide a multi-SRM assessment with differing deployment technicalities Without SRM, variations in the Caribbean’s annual solar resource under an RCP4.5 scenario are negligible when the world attains mean surface temperatures of 1.5 ∘C  and 2.0 ∘C  above pre-industrial values. Further changes in the resource and by extension solar photovoltaic (PV) energy generation due to SRM-induced cooling are also small and do not exceed ± 10% under G4 for either future period. The largest change is observed in May-June-July near Panama. SRM, in comparison, induces significant increases and reductions in wind power generation across much of the examined domain. This provides energy planners with a general perspective on SRM’s potential to be a major factor influencing VRE security planning. Some parts of Central and South America attain increases of 100% and greater, largely under G4cdnc and G4seaSalt. Sections of the Greater Antilles (Lesser Antilles) see mean increases of 50–75% (5–25%) for both 1.5 ∘C  and 2.0 ∘C  years. Marine Cloud Brightening-based SRM (G4cndc and G4SeaSalt) produces the most consistent increases in wind power resources and generation across the Caribbean, compared to G4 SAI-based SRM. This highlights the importance of conducting a multi-SRM assessment. There are also seasonal variations in the SRM-induced changes in both solar and wind resources in the Caribbean. The results suggest that the Caribbean energy sector must actively participate in discussions about the future deployment of SRM globally, given its potential to impact the output of VRE technologies, touted as important to future regional energy security and the development of Caribbean economies.

## Abbreviations

SRM: Solar Radiation Modification
PV: Photovoltaic
CSP: Concentrated Solar Power
VRE: Variable Renewable Energy
RCP4.5: Representative Concentration Pathway 4.5
SIDS: Small Island Developing States
GHG: Greenhouse Gas
SAI: Stratospheric Aerosol Injection
SSI: Stratospheric Sulphate Injection
MCB: Marine Cloud Brightening
GeoMIP: Geoengineering Model Intercomparison Project
